# Perfluorodecaline residue in the anterior chamber of a patient with an intact crystalline lens: a case report

**DOI:** 10.1186/1752-1947-2-13

**Published:** 2008-01-22

**Authors:** Erdinc Aydin, Helin Deniz Demir

**Affiliations:** 1 Department of Ophthalmology, Gaziosmanpasa University School of Medicine, Tokat, Turkey

## Abstract

**Background:**

Perfluorocarbon liquids are frequently used as intraoperative tools in vitreoretinal surgery and may occasionally be retained in the vitreous cavity. We report a patient who underwent pars plana vitrectomy for a giant tear after receiving blunt trauma to his right eye and sustained postoperative perfluorocarbon liquid residue in the anterior chamber in spite of an intact crystalline lens.

**Case presentation:**

Perfluorodecaline was used as a temporary retinal tamponade. Three weeks after the surgery, a residue of heavy liquid was observed in the anterior chamber, even though the patient had an intact crystalline lens without any tilt or dislocation. The remnant of the heavy liquid was taken out of the anterior chamber immediately to avoid secondary complications.

**Conclusion:**

Presence of heavy liquids in the anterior chamber may be associated with zonular defects even though the patient has an intact crystalline lens.

## Background

Perfluorocarbon liquids (PFCLs) with high specific gravity relative to aqueous or subretinal fluid have been frequently used as intraoperative tools and a short-term tamponade in vitreoretinal surgery [1]. It is usually removed at the end of the procedure; however, occasionally residue may be retained due to hazy media. Some clinical reports have been presented in the literature about remnants of perfluorodecaline in the anterior segment [2,3].

We report a case of a patient with perfluorodecaline in the anterior chamber who had an intact crystalline lens following pars plana vitrectomy.

## Case presentation

**Figure 1:**
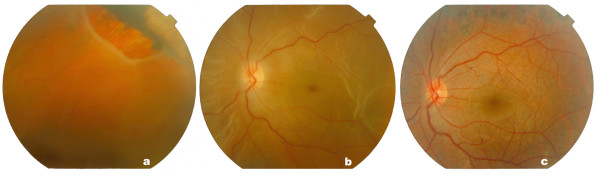
Colour images of the retina before and after operation. (a and b) Giant retinal tear with shallow detached retina involving the macula in the right eye. (c) Reattachment of retina after vitrectomy.

**Figure 2:**
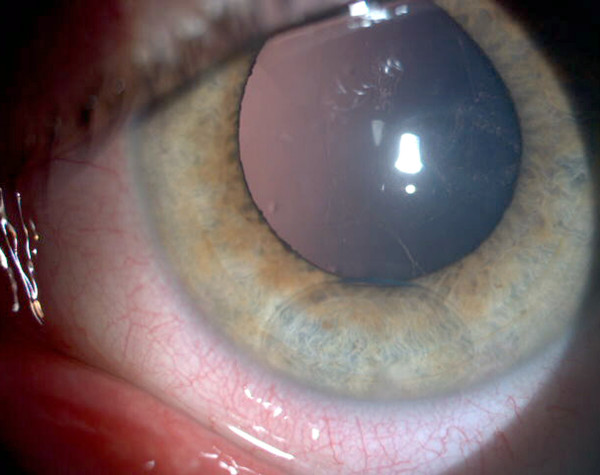
Colour images of perfluorodecalin residue in anterior chamber of a 17-year-old male with an intact crystalline lens.

A 17-year-old male with progressive visual loss and photopsia was admitted to our ophthalmology clinic. He had a history of blunt trauma with a basketball to his right eye 6 months ago and had begun to suffer from visual disturbances since then. His visual acuity (VA) was 20/400 in his right eye. Dilated fundus exam of his right eye revealed a giant tear in the superotemporal retina with a shallow retinal detachment involving the macula (Figure 1a and 1b). His visual acuity was 20/20 and anterior and posterior segments were normal in the left eye. He underwent a three-port pars plana vitrectomy for the giant tear without crystalline lens extraction. Perfluorodecaline was used intraoperatively. Three weeks postoperatively, perfluorodecaline was observed in the anterior chamber in contact with the corneal endothelium (Figure 2). No tilt or dislocation of the crystalline lens was detected on slit lamp biomicroscopy. We referred our patient to another eye clinic for ultrasound biomicroscopic (UBM) or endoscopic examination in order to determine zonular dialysis, but could not obtain these results as the patient refused (4)

After our attempt to remove the perfluorodecaline in the anterior chamber with a 27G needle failed, an anterior chamber wash was performed with balanced salt solution via two wide paracentesis. Two months after vitreoretinal surgery, his best-corrected visual acuity (BCVA) was 40/200 in the right eye. The vision and retina remained stable in his follow-up (Figure 1c).

## Conclusion

Residual PFCLs droplets may pass through inferior iridectomies, and the pupil of aphakic eyes as well as retinal holes if there is residual traction, even though have high surface tension. Their usage as temporary tamponade in human eyes is reported to be well tolerated [4][5]. However, corneal toxicity has also been reported for perfluorooctane, perfluoropolyether and perfluoro-phenanthrene [2,3]. Perfluorodecaline may also have a role in the etiology of some changes, such as corneal oedema and deep corneal vascularization in the area of perfluorodecaline-endothelial contact. It is known that corneal decompensation due to perfluorodecaline-endothelial contact occurs after 4 to 13 weeks, and penetrating keratoplasties can be performed for progressive corneal oedema. Corneal toxicity may be induced by intraocular perfluorodecaline if direct contact is allowed with the corneal endothelium for a period (sometimes as short as 1-month).

Some of these changes may be reversible if perfluorodecaline is aspirated from the anterior chamber [3]. It is assumed that mechanical or the barrier effects of perfluorodecaline may cause the loss of cell density and morphological changes. For these reasons, we decided to remove the perfluorodecaline without any delay.

In the case presented here, we would like to demonstrate that even though the patient has an intact crystalline lens, PFCLs may be postoperatively encountered in the anterior chamber due to zonular defects during vitrectomy surgery or in eyes with trauma.

## Concent

Written consent of the patient was obtained for publication of this case report.

## Competing interests

The author(s) declare that they have no competing interests.

## Authors' contributions

EA wrote up the case and collected the data

HDD supervised management of the case
